# Is the Pattern Changing? Atrial Fibrillation and Screening with Holter Electrocardiograms among Ischemic Stroke Patients in Greenland from 2016 to 2021

**DOI:** 10.3390/jcm12165378

**Published:** 2023-08-18

**Authors:** Nadja Albertsen, Anne Sofie Hansen, Nils Skovgaard, Michael Lynge Pedersen, Stig Andersen, Sam Riahi

**Affiliations:** 1 Department of Geriatric Medicine, Aalborg University Hospital, 9000 Aalborg, Denmark; ansoh@rn.dk (A.S.H.); lasa@rn.dk (S.A.); 2 Department of Clinical Medicine, Aalborg University, 9000 Aalborg, Denmark; sar@rn.dk; 3 Center for Health Research, Ilisimatursarfik (University of Greenland), 3900 Nuuk, Greenlandmilp@peqqik.gl (M.L.P.); 4 Steno Diabetes Center Nuuk, 3900 Nuuk, Greenland; 5 Department of Cardiology, Aalborg University Hospital, 9000 Aalborg, Denmark

**Keywords:** atrial fibrillation, stroke, Greenland, Arctic, Holter, Inuit

## Abstract

A standardized examination regime for ischemic stroke (IS) patients was implemented in Greenland in 2010. Prevalence of atrial fibrillation (AF) of 32% was found among discharged IS patients from 2011 to 2012, and our study aims to estimate the use of Holter ECGs for AF diagnostics and the current prevalence of AF among IS patients in Greenland. Patients discharged from Queen Ingrid’s Hospital in Nuuk between 2016 and 2021 with an ICD-10 diagnosis of IS or stroke without specification were included. Data on Holter recordings, age, gender, medical treatment with rivaroxaban or warfarin, and ICD-10 and ICPC codes for AF were extracted for each patient. The overall incidence of IS from 2016 to 2021 was 133/100,000 and unchanged since 2012. Sixty-eight of the study’s IS patients (14.5%) had AF, and 46% of IS patients with Holter data accessible had a recording according to international recommendations. Our results indicate that fewer IS patients in Greenland have AF than previously. However, the insufficient use of Holter as a diagnostic tool may explain part of the drop, as well as improved preventive treatment with rivaroxaban among AF patients in Greenland. Regardless, IS remains common, and a focus on diagnostics and preventable risk factors should be maintained.

## 1. Introduction

The global incidence of stroke was 12.2 million in 2019, of which 62.4% were IS [1]. Stroke prevalence increases with age, and approximately 42% of all IS occur among those aged 70 years or older [2]. However, although the global incidence of IS is increasing, the global age-standardized incidence is decreasing [3,4]. This change indicates that the incidence is increasing among younger age groups, possibly due to an increasing prevalence of risk factors such as diabetes, high systolic blood pressure and high body mass index (BMI) among the young [2].

Studies have indicated geographical and socioeconomic differences in the incidence of stroke. In the Arctic, a higher incidence of strokes in general has been found among Inuit and Métis populations in Canada compared to the non-native population [5,6], Alaska natives compared to non-natives [7] and among the younger age groups in Greenland compared to Denmark [8]. Similarly, a higher rate of non-hemorrhagic strokes has been found among Native Americans in the US [9].

Patients with atrial fibrillation (AF) are estimated to have a five times higher risk of IS [10] than those without AF, and the risk increases with age [11]. Strokes related to AF carry a higher rate of mortality and disability than those unrelated [12], and notably, the risk of IS is reduced by anticoagulant treatment as evaluated by the CHA_2_DS_2_-VASc score [13].

As with IS, AF is more common among certain ethnic groups when compared to other ethnicities in the same countries [14]. For example, AF is most common among non-Hispanic whites in the US [15,16] and the UK [17], and other studies indicate that AF is more common among the Canadian Métis [6] and young Indigenous Australians [18,19]. However, a study from 2022 found the prevalence of diagnosed AF in Greenland to be at the same level as in other Western countries [20].

It is recommended that IS patients without previously diagnosed AF are screened for AF using a short-term electrocardiogram (ECG) for the first 24 h, followed by at least 72 h of continuous ECG monitoring [21]. In 2010, Greenland implemented a strategy stating that all patients suspected of having suffered an IS should, if possible, be transferred to Queen Ingrid’s Hospital (QIH) in the capital Nuuk for further diagnostics. QIH is the only hospital in Greenland with CT and MR facilities. The recommended examination includes a cerebral scan, a Holter recording, an ultrasound of the carotid arteries, echocardiography and rehabilitation [8].

Following the implementation of this strategy, a study from Greenland from 2013 found that 45 of 139 IS patients from 2011 to 2012 had AF, and most were undiagnosed at the time of hospital admission [22]. The study was based on diagnosis codes on both living and dead patients, and included evaluation of CT scans, medical records, discharge letters and data on Vitamin K treatment from the electronic laboratory system [8,22]. However, the study does not include data on Holter recordings performed on IS patients during this period.

This study has two aims: 1: Estimate the prevalence of AF among patients discharged with an IS diagnosis in Greenland from 2016–2021; and 2: use the number of stroke patients who had a Holter recording performed to evaluate the initiative from 2010 offering all stroke patients in Greenland a standardized examination regime.

## 2. Materials and Methods

### 2.1. Setting

**Figure 1:**
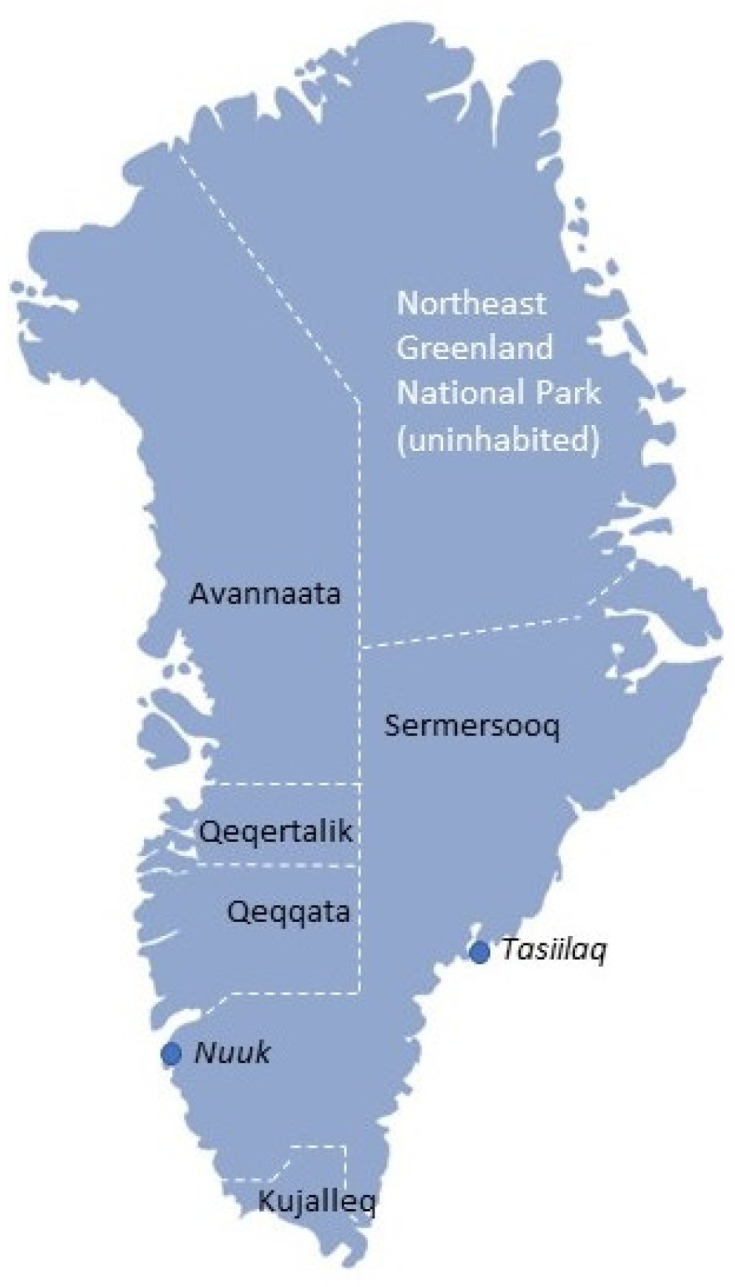
The five administrative regions of Greenland.

Greenland is a self-governing part of the kingdom of Denmark. The population is app. 57,000, of which one-third live in the capital Nuuk. Greenland is divided into five administrative regions (Figure 1).

All towns and most settlements have either healthcare centers or healthcare stations with medically trained staff. Medical doctors are mainly located in larger towns, and telemedicine is widely implemented and used in Greenland. However, transportation of patients is often needed to Nuuk or, in more advanced cases, to Iceland or Denmark. As no towns are connected by roads in Greenland, transportation is patients are mostly carried out by plane or, in some cases, by boat.

Medical examinations and treatment, including pharmaceutical treatment, are free for all residents in Greenland and the distribution of pharmaceuticals in Greenland is handled by the central pharmacy in Nuuk. All prescriptions in Greenland are registered in the medical records by Anatomical Therapeutic Chemical Classification (ATC) codes. At the time of data extraction, two treatments were available for stroke prophylaxis in AF patients in Greenland: the direct-acting oral anticoagulant (DOAC) rivaroxaban and the vitamin K-antagonist warfarin. Both types of medication are also used to treat other conditions than AF, but are used as a proxy for AF treatment in this manuscript, as carried out in a previous paper [20], although with a risk of overestimating the prevalence of AF.

### 2.2. Data Extraction

Data for patients diagnosed in 2016–2020 were extracted in April 2021, and data for patients diagnosed in 2021 were extracted in June 2022. Patient records were not accessed directly, and all data were extracted in pseudonymized form by either research or medical staff in QIH.

Diagnosis codes are registered in the electronic medical record (EMR) system (Cambio COSMIC) upon discharge in Greenland, and the time of discharge and time of diagnosis are therefore used synonymously in the manuscript.

Ischemic stroke: Patients discharged after hospital admission in Nuuk for either IS or stroke without specification from 2016 to 2021 were identified in the EMR system using the International Classification of Disease (ICD), version 10 codes I63* (cerebral infarction) and I64.9 (cerebral stroke without specification [23]). Diagnosis code and year of discharge were extracted for analysis.

Atrial fibrillation: ICD-10 and International Classification of Primary Health Care (ICPC) codes for AF (I48.0, I48.1, I48.2, I48.3, I48.4, I48.9 and K78) were used to identify IS patients with one of these arrhythmias. A patient’s previous diagnosis codes are repeated at discharge if considered relevant for IS diagnosis, meaning that a previous given diagnosis code for an arrhythmia would be repeated. As diagnosis codes are not registered at admission and we had no direct access to the patient records, it was not possible to establish whether the arrhythmia diagnosis was new or old.

Holter recordings: All Holter recordings performed in Greenland are analyzed and stored on a single computer in the outpatient clinic in the Department of Medicine in QIH in the program RTSoft Ultima by Novacor^®^. If the IS patient had a Holter recording in the system, the report was extracted for analysis. If the patient had more than one Holter recording, all reports were extracted. If the recordings were initiated within 72 h after the completion of the previous recording, the recording times were added and registered as the total recording time for that patient. If more than 72 h passed, the recordings were registered as individual recordings. As our data did not include information on the exact date of discharge of the IS patient, we were only able to compare year of discharge and year of Holter recording when estimating the amount of time between the two.

The RTSoft Ultima computer was inaccessible to the research team in 2022 due to a change of location of the team to Denmark, meaning that Holter recordings performed on patients discharged in 2021 were not included in the analysis. The proportion of patients with AF was therefore less certain for 2021 than in the previous years.

Descriptive data and anticoagulation therapy: Data on age at the time of discharge, year of discharge and gender were extracted from the EMR; data on anticoagulation therapy with either rivaroxaban or warfarin at the time of discharge were extracted from the prescription module of the EMR using ATC-codes B01AF01 and B01AA03. The EMR has only recently been expanded with a module for the CHA_2_DS_2_-VASc score, and as we had no access to the patient records, no additional data, including the CHA_2_DS_2_-VASc score, were extracted.

### 2.3. Analysis

The study was conducted as a cross-sectional study. All data were entered into RedCap [24,25] and exported to STATA (StataCorp. 2019. Stata Statistical Software: Release 16. College Station, TX, USA: StataCorp LLC) for statistical analysis. Continuous data were tested for normal distribution using histograms and described using means and standard deviation (SD). Differences between groups were tested using Student’s t-test. Binomial data were compared between groups using Pearson’s chi-squared test. A *p*-value less than 0.05 was considered significant. Incidence rates with 95% confidence intervals (CIs) were calculated using the population size in Greenland from 1 January 2016 to 31 December 2021.

NA visually evaluated all summaries and ECG extractions from all available Holter recordings. When in doubt about whether AF was present or not, Holter recordings were evaluated by a consultant cardiologist (SR).

## 3. Results

### 3.1. IS Incidence

**Figure 2:**
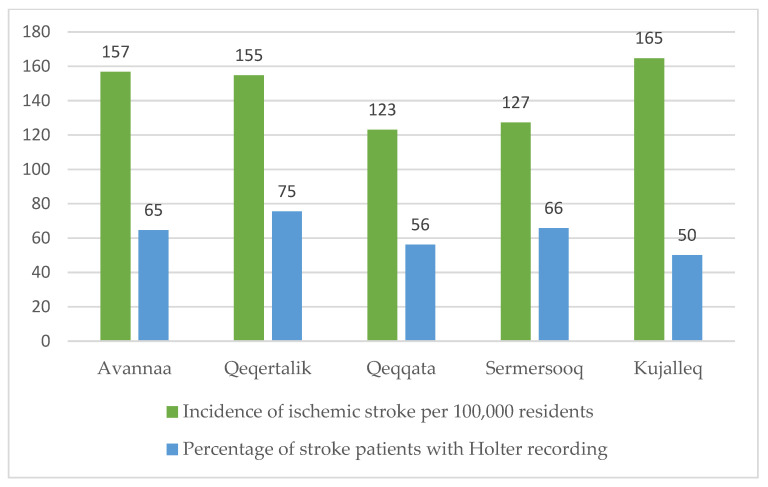
Average incidence of IS per region from 2016 to 2021 and percentage of discharged IS patients with a Holter recording per region from 2016 to 2020.

A total of 470 patients were discharged from QIH from 2016 to 2021 with a diagnosis of IS, corresponding to an incidence of 133 per 100,000 persons (CI 120–147). The incidence was significantly lower in the Qeqqata region when compared to Kujalleq (*p* = 0.016), and in Sermersooq when compared to Kujalleq (*p* = 0.030) (Figure 2, left bars).

Forty-five per cent of the patients were women, and the mean age was 60.9 years (SD 13.1) for women and 61 years (SD 11.1) for men. Sixty-one percent of the women and sixty-three percent of the men were younger than 65 years at the time of diagnosis (Table 1).

For the distribution of ICD-10 diagnosis codes for IS and AF, see Supplemental Table S1.

### 3.2. Holter Recordings

Eighty-three patients were discharged in 2021, and data on the Holter recordings performed on these patients were not accessible to the research team. Of the remaining 387 patients, 245 (63%) had at least one Holter recording registered. Nineteen patients had two Holter recordings and two patients had three recordings. All recordings had been initiated within 72 h after completion of the previous recording. Fifty-seven recordings were double-checked by the consultant cardiologist to verify whether AF was present or not. For conclusions, see Supplemental Table S2.

Eighty-nine per cent (218) of the Holter recordings were performed within the same calendar year as the stroke diagnosis was given, two per cent were performed in the following year and two per cent were performed two years after. The remaining seven per cent were performed in a calendar year one to five years before the year of discharge. The percentage of stroke patients who had had a Holter recording ranged from 50% in the Kujalleq region to 75% in Qeqertalik, as shown in Figure 2, right bars.

The percentage of stroke patients with a Holter recording dropped from 88% in 2016 and 85% in 2017 to 27% and 17% in 2018 and 2019, respectively, followed by an increase to 85% in 2020. The mean recording time with the Holter recorder was 4.0 days (5740 min, SD 2311), the shortest recording was 59 min long and the longest was 10.6 days (15,332 min). The mean analyzable recording time was 2.9 days (4129 min, SD 2230). Sixty-nine per cent of Holter recordings were at least three days long. There were no differences between men and women, nor young and old (65+), regarding the presence of a Holter recording (*p* = 0.471 and *p* = 0.252)

Table 1 shows stroke patients grouped by age, whether they have AF, a Holter recording and treatment with either rivaroxaban or warfarin.

### 3.3. AF among IS Patients

**Figure 3:**
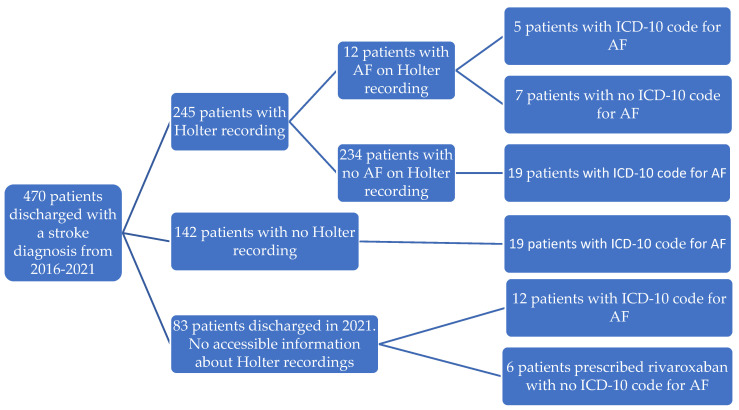
Number of ischemic stroke patients with Holter recording (second row) and AF identified by either diagnosis code, Holter recording or medication (left row).

In total, 68 patients (14.5%) had AF either on the Holter recording, by diagnosis code or by medication (Figure 3).

There was no statistically significant difference between the ratio of men and women with AF, neither in total (*p* = 0.247) nor in the four age groups in Table 1 (*p*-values between 0.077 and 0.414).

Overall, the ratio of stroke patients with AF increased significantly after the age of 65 years (*p* = 0.007). Among women, there was a significant increase in the number of stroke patients with AF from those aged between 65 and 74 years to those aged 75 years or older (*p* = 0.017) and when comparing those younger than 65 years with those older (*p* = 0.011). Among men, there was no significant increase in the ratio between any age group (*p*-values between 0.178 and 0.421).

Finally, 54 (80%) of the patients with AF were treated with either rivaroxaban or warfarin. Of the remaining 14, 8 had a diagnosis code for AF. No patients were treated with warfarin without an AF diagnosis, whereas three women and three men were treated with rivaroxaban but not diagnosed with AF. These six patients were discharged in 2021.

## 4. Discussion

By employing diagnosis codes, ATC codes for anticoagulation therapy and Holter recordings, we conducted a study to determine the prevalence of AF at discharge among IS patients from 2016 to 2021. Our findings revealed that the proportion of IS patients with AF was lower than the reported incidence of 32% in 2011–2012 as reported in Bjørn-Mortensen et al.’s study [22]. The prevalence of AF increases with age, a trend also found in Greenland, where the estimated prevalence of AF among those younger than 60 years is 0.6% and increases to 11.9% among those older than 70 years [20]. Also, AF is more common in strokes with increasing age and has been associated with as many as 36% of strokes among 80 to 89 year olds, compared to 6.7% among the 50–59-year-olds [11] and more than 50% of IS among AF patients older than 90 years [26]. It could therefore be speculated that age difference causes the difference between our studies; however, the patients in Bjørn-Mortensen et al.’s study had a median age of 62 years, only one year older than the mean age of the patients in our study [22].

As in our study, Bjørn-Mortensen et al. used ICD-10 codes to identify stroke patients. They also had access to the patients’ medical records and cerebral scans, allowing them to verify diagnoses and identify diagnosed AF not registered by an ICD-10 code. Eighty-six per cent of the stroke patients in our study had the ICD-10 code I64.9, “stroke, not specified as haemorrhage or infarction”. There might therefore be a risk that we have included some hemorrhagic stroke patients who are less likely to have AF than patients with IS. However, in Mortensen et al.’s study, 90.6% of IS patients had an I64 diagnosis code, and they verified the ICD-10 codes with the CT scans performed on the patients [8].

A shift in prophylactic stroke treatment among AF patients since 2012 may offer some explanation for the drop in included patients with AF. Warfarin was the recommended prophylactic treatment against IS for AF patients in Denmark and Greenland until the indication was added to rivaroxaban and other DOACs in 2012 [27]. Studies have consistently shown that rivaroxaban is at least as effective as warfarin regarding preventing IS [28,29,30], and rivaroxaban has fewer drug interactions and does not need monitoring and changing doses as warfarin does [29]. The dose of warfarin is monitored using INR, and patients are encouraged to maintain an INR in the therapeutic range between 2.0 and 3.0 at least 65% of the time for optimal stroke prevention [31]. INR requires close monitoring, which can be challenging in Greenland, where weather and infrastructure easily affect the transportation of medical equipment, including equipment used for measuring INR. In Bjørn-Mortensen et al.’s study, 84% of patients with AF were treated with an anticoagulant and all with warfarin [22]. In contrast, 79% of AF patients in our study were treated with rivaroxaban and only 1% with warfarin. INR levels are not mentioned in Bjørn-Mortensen et al.’s study, but there may have been some undertreated patients; however, this remains speculative.

As touched upon in the introduction, the CHA_2_DS_2_-VASc score is used to estimate the risk of thromboembolic events among AF patients. The CHA_2_DS_2_-VASc score is based on age, gender and the patient’s history of congestive heart failure, hypertension, thromboembolism/stroke/transient ischemic attack, peripheral vascular disease and diabetes [13]. Patients with a score of zero (men) or one (women) are considered at low risk and are generally not recommended anticoagulation therapy. As our data only include age and gender, the CHA_2_DS_2_-VASc score is not included in our study, nor in Bjørn-Mortensen et al.’s study from 2013 [8]. However, 20% of the patients with AF in our study are not undergoing anticoagulation therapy. The most likely reasons are a low risk of thromboembolic events as indicated by the CHA_2_DS_2_-VASc score (a score below one for men and two for women), contraindications against anticoagulation therapy, patient’s refusal, or undertreatment. A study from 2022 by Albertsen et al. found that 105 out of 790 patients with AF in Greenland were not treated with an anticoagulant and that 77 of those patients had a CHA_2_DS_2_-VASc score where anticoagulation therapy was recommended if no contraindications were present [20]. However, as in this current study, the study from 2022 did not include contraindications for anticoagulation therapy, and any conclusion regarding undertreatment must remain speculative.

Bjørn-Mortensen et al. did not report the number of stroke patients who had Holter monitoring during or after admission in 2011–2012, despite the standardized examination strategy being implemented in 2010. In our study, 63% of patients had a recording performed, and 8% of the remaining had an ICD-10 code for AF, suggesting that they had been diagnosed either before admission or by a short-term ECG during admission (Figure 3). However, 11% of the recordings were performed before or after the year of discharge, meaning that nearly half of the stroke patients are missing a recording performed at least the same calendar year as the stroke diagnosis was given. In addition, 31% of the Holter recordings were shorter than the recommended minimum length of 72 h of continuous monitoring for patients with cryptogenic stroke [21,32], meaning that out of 368 patients eligible for a Holter recording from 2016 to 2020, 169 patients (46%) had a recording in accordance with international guidelines. Holter recordings were only performed in QIH in the Sermersooq region until 2021, when implementation in Ilulissat in Avannaata begun. However, the number of patients screened with a Holter monitor was not higher in this region than elsewhere (*p* > 0.05), indicating that geographical access to Holter screening is not the issue. In Denmark, the recommended percentage of patients screened for AF within four weeks after hospitalization is 75% [33], and the percentage of patients in Greenland screened by Holter monitoring in the same calendar year as discharge was above 80% in 2016, 2017 and 2020. The drop in 2018 and 2019 in Greenland has been discussed with the consultant cardiologist in QIH, and a possible explanation is a temporary lack of Holter equipment. Therefore, insufficient Holter monitoring may explain at least part of the drop of identified AF in IS patients found in our study.

If the ratio of IS patients with AF has dropped, as suggested by our results, the number of non-AF-related IS must have increased since 2012. Based on information from the Global Burden of Disease (GBD) database, Fan et al. suggested that the five most significant metabolic risk factors and two most significant behavioral risk factors for IS are smoking, a diet high in sodium, BMI, high systolic blood pressure, high LDL cholesterol, kidney dysfunction and high fasting blood glucose. They predict that the number of IS deaths from these seven risk factors alone will increase from 3.29 million to 4.90 million by 2030. However, the risk is most pronounced in areas with a low socio-demographic index [3]. The prevalence of most of the risk factors mentioned by Fan et al. is increasing in Greenland. For example, 7.9% of Greenlanders aged 20 years or older had a diagnosis code for hypertension in 2022, and the prevalence of Greenlanders treated with antihypertensive medication had increased from 16.7% in 2012 to 17.5% in 2022 [34]. The number of Greenlanders with a BMI above 30 increased from 13% and 12% among men and women in 1993 to 24% and 32% in 2018 [35], respectively, and although the number of daily smokers is decreasing, 52% of the Greenlandic population were daily smokers in 2018, with the highest number found among men younger than 24 years [35]. Additionally, the diet in Greenland has been changing from a traditional, mainly animal-based diet to including a higher rate of imported foods and more food containing sugar and saturated fat [35,36]. Regarding low-density lipoprotein (LDL) cholesterol, a specific variant of the gene coding for the LDL receptor called p.G137S is only found in Arctic populations and is carried by 29.5% of the Greenlandic population. The effect size of the gene has been estimated to be 0.75 mmol/L (0.66 SD) per allele, and 54.7% of homozygous carriers in Greenland fulfil the criteria for familial hypercholesterolemia [37]. In 2022, Bundgaard et al. found that among 13,895 adult Greenlanders with a lipid profile, 79.4% had dyslipidemia, and among those with elevated LDL cholesterol, only 19% were treated with statin [38]. Finally, approximately 9% of the Greenlandic population aged 18 years or older have diabetes [39], of which 18% are estimated to be associated with two genetic variants: the HNF1A affecting the maturity-onset diabetes of the young (MODY) gene [40] and the TBC1DA variant, affecting insulin-mediated glucose uptake in the muscles [41].

### Limitations

The main limitation of this study is that it is based on diagnosis codes only registered upon hospital discharge from QIH in Nuuk. The study, therefore, excludes patients that were not transferred to QIH or died before or during hospital admission. However, the registrations of cardiovascular diagnoses in Greenland are generally valid [42], and the risk of missing discharged patients with an appropriate stroke diagnosis is therefore low in our study.

Using rivaroxaban and warfarin as a proxy for AF risks overestimating the prevalence of AF as these medications are also used preventively in other conditions, such as artificial heart valves or deep venous thrombosis. However, we only found IS patients without an AF diagnosis treated with rivaroxaban or warfarin in 2021, indicating that any overestimation is limited.

We could not see if the patients had already been diagnosed with AF on a short-term ECG upon admission or if they were diagnosed with AF before their admission, which could explain why Holter monitoring was not performed. Additionally, we were not able to see if the patients were treated with an anticoagulant prior to their admission for IS. The ratio of pre-stroke AF and post-stroke AF is therefore unknown in our study.

Our study does not include time from hospital discharge to initiation of a Holter recording. As described in the discussion, Holter recordings were only performed in QIH until 2021, so it should be expected that patients from other regions had a recording performed during their admission for IS, as it may be considered too costly and time-consuming to fly them back to Nuuk for this simple examination. However, as we have chosen to describe the times of Holter recordings by calendar year, some patients discharged in January one year may have had a recording performed in December and our results incorrectly indicate a considerable time gap between IS and Holter recording.

In addition, the presence of AF may have been underestimated in 2021 due to the lack of access to Holter recordings. Also, conducting repeated monitoring could have potentially revealed a higher incidence of AF.

Incorporating long-term monitoring methods such as 7- or 14-day patch monitors, implantable monitors and smartwatches can offer a higher diagnostic yield when it comes to detecting and documenting AF. These monitoring techniques provide extended periods of observation, enabling more comprehensive and accurate identification of AF episodes [43]. However, due to the retrospective design of this study, the opportunity for such repeated monitoring was not available.

As mentioned earlier, our data do not allow for a complete calculation of the CHA_2_DS_2_-VASc score and it is therefore not included in our study. Similarly, it was not included in the study from 2013 [8]. However, it would be relevant to consider that the risk factor profile among AF patients in Greenland may have changed since 2011 and 2012.

Our study does not include any information on the ethnicity of the participants. As mentioned above, the Greenlandic Inuit population have specific genetic characteristics that may affect their risk of stroke. However, we cannot apply these data to our findings on the incidence of IS or AF.

The EMR used in Greenland is not fully implemented in the town Tasiilaq (Figure 1) on the east coast. However, it is used when patients from Tasiilaq is admitted to QIH, and patients from Tasiilaq diagnosed with IS in QIH are therefore included in the study.

## 5. Conclusions

The data presented suggest a potential decline in the proportion of ischemic strokes associated with AF in a specific population compared to previous years. However, it is important to note that direct comparative data, such as the CHA_2_DS_2_-VASc score, is required to establish this point. Furthermore, the observed trend may be influenced by the use of anticoagulation therapy in patients with confirmed AF, although additional information about the characteristics of the studied population is needed to confirm this hypothesis. Our results indicate that more than half of the patients lack sufficient continuous ECG monitoring, and AF among the IS patients may therefore be underdiagnosed. Strategies for improving the diagnostics among IS patients should be considered, as treatment with the correct anticoagulant after the stroke is vital. However, an increasing number of IS may be caused by other potentially preventable risk factors than AF.

## Figures and Tables

**Table 1:** Discharged IS patients by age groups, AF and anticoagulation therapy.

Age	<55 Years		55–64 Years		65–74 Years		75+ Years		Total	
	Women	Men	Women	Men	Women	Men	Women	Men	Women	Men
Stroke, n (% of all patients) ^a^	58 (27)	65 (25)	72 (34)	97 (38)	43 (20)	72 (28)	40 (19)	23 (8.9)	213 (45)	257 (55)
AF, n (% of patients in age group) ^a^	2 (3.4)	7 (10.8)	8 (11.1)	15 (15.5)	4 (9.3)	16 (22.2)	13 (33)	3 (13)	27 (12.7)	41 (16.0)
Holter, n (% of patients in age group) ^b^	34 (63)	37 (64)	34 (63)	44 (59)	22 (69)	43 (70)	21 (62)	10 (53)	111 (64)	134 (63)
Rivaroxaban, n (% of patients with AF in age group) ^a^	2 (100)	7 (100)	7 (87.5)	12 (80)	4 (100)	9 (56.3)	9 (69.2)	3 (100)	22 (81.5)	31 (75.6)
Warfarin, n (% of patients with AF in age group) ^a^	0 (0)	0 (0)	0 (0)	0 (0)	0 (0)	1 (6.3)	0 (0)	0 (0)	0 (0)	1 (2.4)

^a^ Includes discharged patients from 2016 to 2021. ^b^ Discharged stroke patients from 2021 not included (Holter recordings not available).

## Data Availability

Data supporting the findings of this study are available from the corresponding author upon reasonable request.

## Supplementary Materials

The following supporting information can be downloaded at: https://www.mdpi.com/article/10.3390/jcm12165378/s1, Table S1: ICD-10 codes stroke and AF; Table S2: Conclusions of the Holter revisions.
