# Supplementary material for: Is the Pattern Changing? Atrial Fibrillation and Screening with Holter Electrocardiograms among Ischemic Stroke Patients in Greenland from 2016 to 2021
Source: J Clin Med. 2023 Aug 18;12(16):5378. doi: 10.3390/jcm12165378 (PMC10455734; doi:10.3390/jcm12165378)
Supplement: Supplementary file 1 [file jcm-12-05378-s001.zip › jcm-2497621-supplementary.pdf]

Table S1: ICD-10 codes stroke and AF

**ICD10-code stroke, n (%)***	**Women (n=244)**	**Men (n=289)**	**Total (n=533)**
I63	6 (2.5)	17 (5.9)	23 (4.3)
I63.0	1 (0.4)	1 (0.3)	2 (0.3)
I63.3	3 (1.2)	2 (0.6)	5 (0.9)
I63.4	1 (0.4)	1 (0.3)	2 (0.3)
I63.8	0 (0)	1 (0.3)	1 (0.2)
I63.9	16 (6.6)	34 (11.7)	50 (9.4)
I64.9	188 (77.0)	214 (74.0)	402 (75.4)
**ICD-code atrial fibrillation, n (%) ** **	**Women (n=22)**	**Men (n=33)**	**Total (n=55)**
I48.0	15 (68.2)	15 (45.5)	30 (54.5)
I48.1	1 (4.5)	2 (6.1)	3 (5.5)
I48.2	1 (4.5)	3 (9.1)	4 (7.3)
I48.3	0 (0.0)	0 (0.0)	0 (0.0)
I48.4	0 (0.0)	0 (0.0)	0 (0.0)
I48.9	8 (36.4)	12 (36.4)	20 (36.4)
K78	5 (22.7)	7 (21.2)	12 (21.8)

* Thirty patients had been given more than one stroke diagnosis.

** Eleven patients had more than one ICD 10 or ICPC code for AF.

Table S2: Conclusions of the Holter revisions

**Conclusion**	**Number of patients**
Sinus rhythm	8
Atrial Fibrillation	7
Focal atrial tachycardia	19
Intermittent nodal rhythm	5
Supraventricular extrasystoles	6
Intermittent pace rhythm	1
Mb Wenkebach	1
Noise	10
**Total**	**57**
